# General Figures of Merit *ZQ* for Thermoelectric Generators Under Constant Heat‐In Flux Boundary

**DOI:** 10.1002/advs.202303695

**Published:** 2023-09-26

**Authors:** Huan Li, Yupeng Wang, Kang Zhu, Zhijia Han, Xinzhi Wu, Shuaihua Wang, Wenqing Zhang, Weishu Liu

**Affiliations:** ^1^ Department of Materials Science and Engineering Southern University of Science and Technology Shenzhen Guangdong 518055 P. R. China; ^2^ Guangdong Provincial Key Laboratory of Functional Oxide Materials and Devices Southern University of Science and Technology Shenzhen Guangdong 518055 P. R. China

**Keywords:** constant heat‐in flux, figures of merit, optimization strategies, thermoelectric generators, thermoelectric materials

## Abstract

The thermoelectric figure of merit *ZT* bridges the efficiency and material parameters for a thermoelectric device operating under constant temperature of the hot‐ and cold‐source thermal boundary (Type‐I TB). However, many application scenarios fall under the constant heat‐in flux (*q*_h_) and constant cold‐source temperature (*T*_c_) thermal boundary (Type‐II TB), for which a figure of merit is absent for more than half a century. This study aims to fill this gap and propose a figure of merit *ZQ*_D_ for the thermoelectric devices under the Type‐II TB condition, defined as $ZQ_{D}=\left(\frac{ZT_{c}}{ZT_{c}+1}\right)\left(\frac{h}{\kappa }\right)\left(\frac{q_{h}}{T_{c}}\right)$, where *Z*, *h*, and *κ* are the traditional figure of merit, leg height, and thermal conductivity, respectively. The effectiveness of *ZQ*_D_ is verified through both numerical calculations and experiments, which are more accurate and practical than *ZT*. Furthermore, a system‐level figure of merit *ZQ*_S_ is suggested after considering the external thermal resistance. Finally, optimization strategies for thermoelectric systems based on *ZQ*_S_ are proposed, showing a 30% enhancement in the efficiency. *ZQ*_D_ and *ZQ*_S_ are expected to be widely used in the thermoelectric field.

## Graphical abstract

To fill the absence of a figure of merit suitable for thermoelectric generators operating under the Type‐II thermal boundary, this work proposes new indicators (*ZQ*) by analytically solving the fundamental transport equation, which gives a clear physical picture behind the relationship between generation efficiency, materials properties geometrical parameters, and operating conditions.

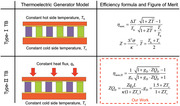

## 1 Introduction

Thermoelectric generators (TEGs), which can convert heat directly into electricity based on the Seebeck effect,^[^ ^1^ ^]^ are not only promising for waste heat harvesting from vehicles, but are also inspiring new applications in the Internet of Things^[^ ^2^ ^]^ and wearable electronics.^[^ ^3^ ^]^ As the cornerstone of TEG, Ioffe's work^[^ ^4^ ^]^ connects the TEG efficiency with the figure of merit *ZT* (*ZT* = *S* ^2^ *σT* *κ* ^−1^, where *S*, *σ*, *κ*, and *T* are the Seebeck coefficient, electrical conductivity, thermal conductivity, and temperature, respectively). The convenience of using the figure of merit *ZT* as a thermoelectric device performance index has been a consensus in the thermoelectric (TE) field. Several modified thermoelectric figures of merit^[^ ^5^, ^6^ ^]^ and efficiency formulae^[^ ^7^, ^8^ ^]^ have been proposed that consider the effects of temperature‐dependent material properties, contact interface, etc.

**Figure 1:**
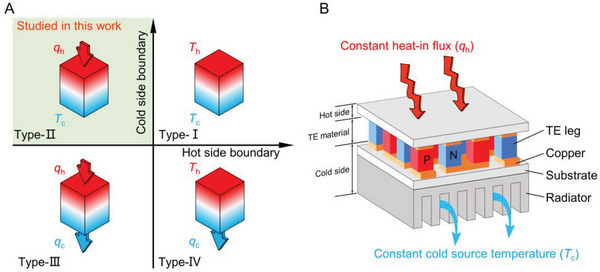
A) Four typical thermal boundary conditions in TEG application (boundary in the second quadrant was studied) and B) typical TE system under constant heat‐in flux and cold source temperature thermal boundary (Type‐II TB).

The thermal boundaries (TBs) of the TEG include fixed temperature (Dirichlet boundary), fixed heat flux (Neumann boundary), and fixed convective heat transfer coefficient (Robin boundary).^[^ ^9^ ^]^ The latter can be simplified as a special Dirichlet boundary when considering it as the thermal resistance between the TEG and the environment. According to the different positions of TBs, there are four working modes of TEG (**Figure** **1**A), such as the fixed heat source temperature (*T*_h_) and cold source temperature (*T*_c_) (Type‐I TB), and fixed heat‐in flux (*q*_h_) and *T*_c_ (Type‐II TB). The TB condition for the derivation of *ZT* is Type‐I TB. However, this type of TB condition is inapplicable in many application scenarios, including solar thermoelectric generators (STEG),^[^ ^10^ ^]^ radioisotope thermoelectric generators (RTEG),^[^ ^11^ ^]^ and recovery of radiant heat from silicon casting.^[^ ^12^, ^13^, ^14^ ^]^ Ordinarily, if the temperature of a heat source changes much more quickly than the heat flux, it is better to use the Type‐II TB model to evaluate their efficiencies.^[^ ^15^ ^]^ For ideal TEG operating under the Type‐II TB, the hot side temperature of TEG changes with the load resistance, resulting in the voltage–current curve being non‐linear. So, the optimal load resistances for the maximum output power and efficiency of Type‐II TB are significantly different from that of Type‐I TB. Moreover, owing to the *ZT* derived under the Type‐I TB condition, the feasibility of the *ZT* and reasonability of the optimization strategies concluded from the *ZT* are questionable for the TEG working in the Type‐I TB condition. Therefore, a new figure of merit suitable for the Type‐II TB conditions is required to directly reflect the power‐generation performance in relevant applications.

To achieve this, a bottom‐up theoretical analysis is required to illuminate the relationships between the TEG performance (i.e., maximum output power and efficiency) and relevant parameters (including material properties, structural parameters, and external thermal conditions), as in the Ioffe, but under different thermal boundaries. The earliest study on this analytical model was reported by Castro et al.^[^ ^16^ ^]^ in 1960. Thacher then derived new equations for optimum load resistance considering the influence of the Thomson effect and heat loss based on Raag's work.^[^ ^17^ ^]^ However, these were all blocked midway due to highly nonlinear and complicated analytical derivations. Gao^[^ ^15^, ^18^ ^]^ made significant progress in analytical solving using the average optimized load ratio *m*_ave_ between *m*_v_ under the condition *V* = *V*_o_/2 and *m*_I_ under the condition *I* = *I*_Q_/2 (where *V*_o_ and *I*_Q_ are the open‐circuit voltage and shortage current, respectively). Notably, the use of the two sets of equations renders the final expression of the maximum efficiency too complex to reveal the direct relationship between the efficiency and parameters (Note S1, Supporting Information, Reduction method of Gao's model for details). Using another reduction method, Apertet et al.^[^ ^19^ ^]^ proposed an optimized load ratio *m*_opt_ under the Type‐II TB condition according to the force–flux formalism and the corresponding maximum efficiency formula can be derived as:

(1) $$\left\{\begin{array}{cc} \eta_{\max}=\frac{ZR_{t}Q_{h}}{4\left(1+ZT_{\mathrm{ave}}\right)} & \left(a\right)\\ m_{\mathrm{opt}}=1+ZT_{\mathrm{ave}} & \left(b\right) \end{array}\right.$$

where *R*_t_ is the thermal resistance of the TE leg, *Q*_h_ is the heat input power, and *T*_ave_ is the average of *T*_h_ and *T*_c_. Nevertheless, the simplification incurs a cost of precision because it neglects the Joule effect and assumes that the heat flux input is equal to the output. Alternatively, many efforts have been made to search for computer simulation methods,^[^ ^20^, ^21^, ^22^ ^]^ which can provide a relatively accurate result; however, they lose the physical picture behind the TEG performance.

Here, we proposed an engineering figure of merit *ZQ*_D_ for TE devices under the Type‐II TB condition by solving the 1D transport equation of the thermoelectric conversion process. Furthermore, we derived the system‐level efficiency formula under the Type‐II TB condition and the corresponding figure of merit *ZQ*_S_, which have a similar form to the device‐level efficiency. The effectiveness of the relationship between the figure of merit *ZQ*_S_ and efficiency is discussed and experimentally verified, showing high consistency with numerical calculations.

## 2 Results and Discussion

### 2.1 Figure of Merit *ZQ*_D_ of TE Devices Under Type‐II TB Condition

A typical TE system of the Type‐II TB condition is shown in Figure 1B. A heat exchanger is adopted at the cold side, acting as a heat path between the thermoelectric module and surroundings. This section only focuses on the TE material part of the TE system. A comprehensive analysis has been detailed in the next section to handle the whole TE system, including both the TE material and heat exchanger. For convenience, the word “efficiency” mentioned later refers to the “maximum generation efficiency”. The efficiency of the TE devices under the Type‐II TB condition based on the governing equation is derived as (Note S2, Supporting Information; Derivation of the maximum generation efficiency formula for the TE device model under the Type‐II TB condition for a detailed derivation):

(2) $$\left\{\begin{array}{cc} \eta_{\max,D}=\frac{1}{g_{D}}\frac{\sqrt{g_{D}\cdot ZQ_{D}+1}-1}{\sqrt{g_{D}\cdot ZQ_{D}+1}+1} & \left(a\right)\\ m_{\mathrm{opt},D}=ZT_{c}+1 & \left(b\right)\\ g_{D}=1+\frac{1}{2m_{\mathrm{opt},D}} & \left(c\right)\\ ZQ_{D}=\left(\frac{ZT_{c}}{ZT_{c}+1}\right)\left(\frac{h}{\kappa }\right)\left(\frac{q_{h}}{T_{c}}\right) & \left(d\right) \end{array}\right.$$

where *T*_c_ is the cold source temperature, *h* is the leg height, and *g*_D_ falls into a narrow range (≈1.2–1.5) for the majority of the reported TE materials, as shown in Figure S1 (Supporting Information). *ZQ*_D_ is a dimensionless parameter, *m*_opt_ is the optimal ratio of the load resistance to the electrical resistance of the TE leg for maximum generation efficiency, and *q*_h_ is the heat flux (*q*_h_ = *Q*_h_ *A* ^−1^, where *A* is the cross‐sectional area of the TE leg). *ZQ*_D_ is related to the material physical properties (*S*, *σ*, and *κ*), structural parameter (*h*), and external thermal conditions (*q*_h_ and *T*_c_). Notably, *q*_h_ and *h* always exist in the form of *q*_h_ *·h* in both our model (Equation 2) and governing model (Equation S11, Supporting Information). This suggests that *q*_h_ and *h* have equivalent effects in determining the efficiency; therefore, the impact of *q*_h_ *·h* on the efficiency is discussed.

The accuracy of the efficiency formula derived under the Type‐II TB condition was investigated through a direct comparison between the efficiency given by Equation (2) (*η*_D, eq_) and numerically calculated efficiency (*η*_D, num_) by solving the governing equation (Equation S11, Supporting Information) over a broad range of relevant parameters and different materials. In this study, the power factor (*PF* = *S^2^σ*) and *κ* value of some classical TE materials at 300 K are used with a constant properties assumption, including *p*‐SiGe,^[^ ^23^ ^]^ *p*‐MgAgSb,^[^ ^24^ ^]^ *p*‐half‐Heusler,^[^ ^25^ ^]^ *n*‐CoSb,^[^ ^26^ ^]^ *n*‐MgSnGe,^[^ ^27^ ^]^ and *n*‐BaGaSn,^[^ ^28^ ^]^ as shown in Figure S1 (Supporting Information). This assumption is useful and common in the model simplification, just as Ioffe's work.^[^ ^4^ ^]^

**Figure 2:**
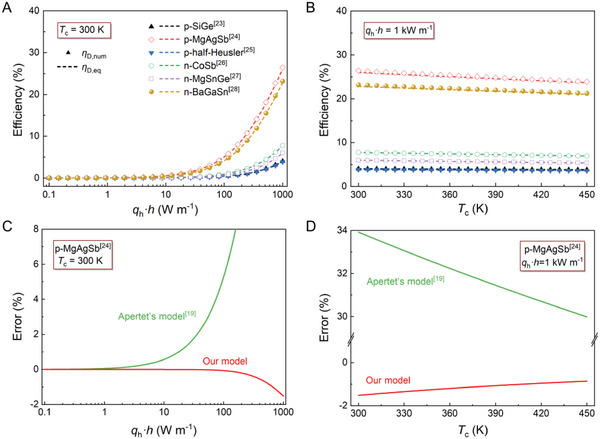
Maximum generation efficiency of TE device model under Type‐II TB condition of six typical TE materials under different: A) heat‐in flux multiplied by height of TE leg (*q*_h_ *·h*), and B) cold source temperature (*T*_c_) Symbols and dotted lines represent maximum generation efficiency numerically calculated from governing equation (*η*_D,num_) and value calculated through our equation (*η*_D,eq_), respectively. Physical properties of TE materials^[^ ^23^, ^24^, ^25^, ^26^, ^27^, ^28^ ^]^ at 300 K were used. Error in TE device model under Type‐II TB condition proposed by Apertet et al.^[^ ^19^ ^]^ and this study, as compared with *η*_D,num_ under different: C) *q*_h_ *·h*, and D) *T*_c_. The red and green lines represent errors calculated using our model and Apertet's models, respectively. Physical properties of *p*‐MgAgSb^[^ ^24^ ^]^ at 300 K were used.

**Figure** **2** shows surprisingly good consistency between the *η*_D,eq_ and *η*_D,num_ within the investigated parameters range, including *q*_h_ *·h* = 0.1–1000 W m^−1^ (Figure 2A) and *T*_c_ = 300–450 K (Figure 2B). *p*‐MgAgSb^[^ ^24^ ^]^ has a high *η*_D,num_ value because of its relatively high *PF* and low *κ* near room temperature (Figure S1, Supporting Information). The relative errors were <2% during the investigation. For comparison, the efficiency calculated using Apertet's model^[^ ^19^ ^]^ was also calculated, with detailed numerical calculations relative to the *T*_h_ shown in Equation (S15) (Supporting Information). Figure 2C,D compares the errors of Apertet's model (Equation 1) and our model (Equation 2) with those of the numerical calculation (Equation S11, Supporting Information). The Apertet's model shows a minor positive error when *q*_h_ *·h* is <10 W m^−1^. However, error significantly increased with higher *q*_h_ *·h*. On the contrary, our model is more effective in a wide range of *q*_h_ *·h*, i.e., in the range of 0.1–1000 W m^−1^ and *T*_c_ of 300–450 K. Furthermore, the increase in the error with the increase in the *q*_h_ *·h* can attribute to the simplification process of Equation (S13) (Supporting Information), which ignores the term of *ZR*_t_ *Q*_h_ (*ZR*_t_ *Q*_h_ = *Zq*_h_ *·h* *κ* ^−1^).

To further understand the influence of material properties (*PF* and *κ*) on the efficiency, the evolution of *η*_D,eq_ and *η*_D,num_ under different *PF* (10^−4^–10 W m^−1^ K^−2^) and *κ* (10^−4^–1 W m^−1^ K^−1^) are investigated. As shown in Figure S2 (Supporting Information), all the *η*_D,eq_ and *η*_D,num_ values tend to specific values when *PF* is infinite or *κ* is infinitesimal. This phenomenon can be explained analytically using Equation (2).

(3) $$\left\{\begin{array}{cc} \lim_{PF\to \infty }\eta_{\max,D}=\frac{\sqrt{q_{h}h+\kappa T_{c}}-\sqrt{\kappa T_{c}}}{\sqrt{q_{h}h+\kappa T_{c}}+\sqrt{\kappa T_{c}}} & \left(a\right)\\ \lim_{\kappa \to 0}\eta_{\max,D}=1 & \left(b\right) \end{array}\right.$$

The limit value of the efficiency for an infinite *PF* is determined by *q*_h_ *·h*, *κ*, and *T*_c_, whereas for an infinitesimal *κ*, it is 1. These results agree with the phenomenon shown in Figure S2 (Supporting Information), which proves the effectiveness of our model.

**Figure 3:**
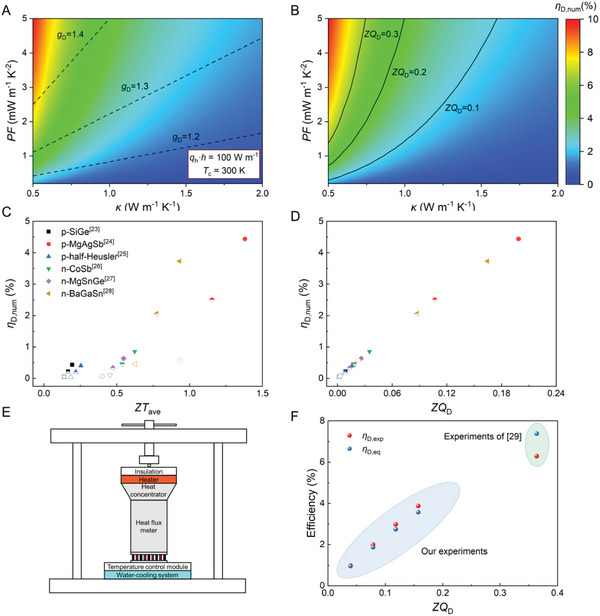
2D plots of efficiency of TE device numerically calculated from governing equation (*η*_D,num_) versus power factor (*PF*) and thermal conductivity (*κ*). Solid and dotted lines represent various contours of A) *g*_D_ and B) *ZQ*_D_ versus *PF* and *κ*, respectively. *η*_D,num_ as a function of: C) *ZT*_ave_ and D) *ZQ*_D_. Physical properties of TE materials^[^ ^23^, ^24^, ^25^, ^26^, ^27^, ^28^ ^]^ at 300 K were used. Various points indicate distinct operating environments, including open symbols *q*_h_ *·h* = 10 W m^−1^ and *T*_c_ = 300 K, half‐up symbols *q*_h_ *·h* = 50 W m^−1^ and *T*_c_ = 350 K, and solid symbols *q*_h_ *·h* = 100 W m^−1^ and *T*_c_ = 400 K. E) Schematic for measuring maximum generation efficiency of TEG under Type‐II TB condition. F) Maximum generation efficiency of TE devices as a function of *ZQ*_D_. *η*_D,exp_ (red dots) and *η*_D,eq_ (blue dots) represent efficiencies calculated from the experiment and proposed equation, respectively.

Then, the feasibility of using the dimensionless parameter *ZQ*_D_ as a figure of merit to reflect the efficiency under the Type‐II TB condition was investigated. First, the superiority of *ZQ*_D_ as an indicator over *g*_D_ was demonstrated. **Figure** **3**A,B shows the 2D plots of *η*_D,num_ versus *PF* (0.2–5 mW m^−1^ K^−2^) and *κ* (0.5–2 W m^−1^ K^−1^) under *q*_h_ *·h* = 100 W m^−1^ and *T*_c_ = 300 K. The contours of *ZQ*_D_ and *g*_D_ versus the same ranges of *PF* and *κ* are also plotted. Evidently, the contours of *ZQ*_D_ almost coincide with the contours of *η*_D,num_, whereas the contours of *g*_D_ show poor consistency with those of the *η*_D,num_. *g*_D_ cannot reflect the variation in the efficiency when the material properties (*PF* and *κ*) change. Moreover, compared with the *ZQ*_D_, the expression of *g*_D_ lacks a description of the boundary condition (*q*_h_), which means that the *g*_D_ cannot reflect the change in the efficiency when the thermal environment changes. Thus, *ZQ*_D_ was selected as a newly defined indicator, and *g*_D_ was used as a correction coefficient. Subsequently, the advantages of *ZQ*_D_ compared with the traditional indicator (*ZT*_ave_) are discussed. *ZT*_ave_ is estimated from *ZT*_ave_ = *Z*(*T*_h_ + *T*_c_)/2, where *Z* is used as the value at 300 K and *T*_h_ is estimated from Equation (S15) (Supporting Information). Figure 3C,D shows the *η*_D,num_ in the Type‐II TB condition as a function of the *ZQ*_D_ and *ZT*_ave_ for selected classical TE materials^[^ ^23^, ^24^, ^25^, ^26^, ^27^, ^28^ ^]^ under three different *q*_h_ *·h* and *T*_c_ conditions, including the open symbols as *q*_h_ *·h* = 10 W m^−1^ and *T*_c_ = 300 K, half‐up symbols as *q*_h_ *·h* = 50 W m^−1^ and *T*_c_ = 350 K, and solid symbols as *q*_h_ *·h* = 100 W m^−1^ and *T*_c_ = 400 K. The *η*_D,num_ has a tight correction with the *ZQ*_D_ in a wide range of *PF*, *κ*, *q*_h_ *·h*, and *T*_c_, showing that a higher *ZQ*_D_ corresponds to a higher *η*_D,num_ in the Type‐II TB condition. By contrast, the correlation between the *η*_D,num_ and *ZT*_ave_ was scattered, especially when comparing the different materials.

Furthermore, we experimentally measured the Type‐II TB conditional efficiency *η*_D,exp_ of a Bi_2_Te_3_ devicewith 18 pairs of legs (1.5 × 1.5 × 2 mm). A steel block was added to the hot side of the TE material to test the heat flux according to Fourier's law. The cold‐side temperature was maintained at a fixed value (300 K) using a thermoelectric device controlled by a PID program and water‐cooling system, as shown in Figure 3E. Different *ZQ*_D_ values were achieved by varying the *q*_h_ *·h*, including 50, 100, 150, and 200 W m^−1^. The transport properties of commercial *n*‐BiTe and *p*‐BiTe used in this study have a relatively weak correlation with the temperature are shown in Figure S3 (Supporting Information). Hence, we use the integral average of the properties to estimate the efficiency and choose an empirical temperature interval of 300–450 K as the upper and lower limits of integration. Detailed descriptions of the measurement steps are presented in Note S3 (Supporting Information), “Measuring the maximum generation efficiency of TE devices and systems under the Type‐II TB condition.” An excellent linear relationship between the Type‐II TB conditional efficiency *η*_D,exp_ and *ZQ*_D_ is presented (Figure 3F). For comparison, the *η*_D,eq_ is also presented, which is slightly <*η*_D,exp_. This may be attributed to the intrinsically negative error of our model, which underestimates theoretical efficiency (Figure 2). Furthermore, the data on the flat‐panel solar thermoelectric generators with high thermal concentration in reference^[^ ^29^ ^]^ is presented, which has a *T*_c_ = 293 K and *q*_h_ *·h* = 361 W m^−1^. The integral average of *PF* and *κ* in the range 300–450 K were used for the reference data. The *η*_D,exp_ versus *ZQ*_D_ follows an approximately linear relationship. Radiant heat loss may be an additional reason for the difference between the *η*_D,exp_ and *η*_D,eq_. Generally, *ZQ*_D_ exhibits good figure of merit for Type‐II TB conditional thermoelectric power generation.

### 2.2 Figure of Merit *ZQ*_S_ of TE Systems Under Type‐II TB Condition

To make the figure of merit more practical, we considered more influential factors, such as the interface thermal resistance, heat sink, and spacing between the TE legs. Interface thermal resistances between the TE devices and heat source are inevitable, which could significantly reduce the effective temperature difference and hence reduce the efficiency. In our previous work,^[^ ^7^ ^]^ we systematically investigated the impact of the thermal resistances on Type‐I TB conditional efficiency and derived a system efficiency formula by introducing a dimensionless thermal resistance parameter *f*_c_ for the cold side and *f*_h_ for the hot side. The dimensionless parameter *f*_c_, which is defined as the ratio of the cold side thermal resistance (*R*_c_) to the thermal resistance (*R*_t_) of the TE leg, has a similar definition applying to the parameter *f*_h_. Notably, the hot side thermal resistance is not considered in the Type‐II TB conditions because the temperature distribution on the hot side does not affect the heat‐in flux of the TEG under the ideal heat transfer process. As a result, only the cold side thermal resistance parameter *f*_c_ is considered. Besides, *f*_c_ also reflects the effect of heat sink thermal resistance and convective heat transfer between the TE system and environments because the convective heat transfer can be regarded as the thermal resistance. Spacing between the TE legs often refers to the fill factor, *F*, which is defined as the ratio of the TE area to the receiver area. The efficiency formula for the TE systems under the Type‐II TB condition (Note S4, Supporting Information; Derivation of the maximum generation efficiency formula for TE systems model under Type‐II TB condition for a detailed derivation) is:

(4) $$\left\{\begin{array}{cc} \eta_{\max,S}=\frac{1}{g_{S}}\frac{\sqrt{g_{S}\cdot ZQ_{S}+1}-1}{\sqrt{g_{S}\cdot ZQ_{S}+1}+1} & \left(a\right)\\ m_{\mathrm{opt},S}=\left(ZT_{c}+1\right)+\frac{f_{c}Zq_{h}h}{\kappa F} & \left(b\right)\\ g_{S}=1+\frac{1}{2m_{\mathrm{opt},S}} & \left(c\right)\\ ZQ_{S}=\frac{ZQ_{D}}{F+f_{c}ZQ_{D}} & \left(d\right) \end{array}\right.$$

Equation (4a) (system efficiency) has the same form as Equation (2a) (device efficiency), but with different parameters *g* and *ZQ*. *g*_S_ (Equation 4c) is slightly <*g*_D_ (Equation 2c) because of the extra term *Zf*_c_ *q*_h_ *h*/(*Fκ*) in the denominator. Hence, the *g*_S_ was approximately constant. The difference between the *ZQ*_S_ and *ZQ*_D_ is expressed as the excess term *f*_c_ *ZQ*_D_, which accounts for the effect of the cold‐side thermal resistance. When *f*_c_ = 0, Equation (4) returns to the device equation, that is, Equation (2).

**Figure 4:**
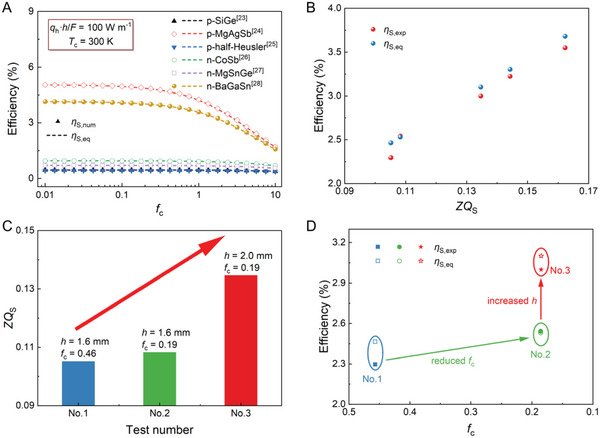
A) Maximum generation efficiency of TE system model under Type‐II TB condition of six typical TE materials^[^ ^23^, ^24^, ^25^, ^26^, ^27^, ^28^ ^]^ under different ratios of cold side thermal resistance to thermal resistance of TE module (*f*_c_). Symbols and dotted lines represent the maximum generation efficiency numerically calculated from the governing equation (*η*_S,num_) and the value calculated through our equation (*η*_S,eq_), respectively. Physical properties of TE materials^[^ ^23^, ^24^, ^25^, ^26^, ^27^, ^28^ ^]^ at 300 K were used. B) Maximum generation efficiency of TE systems as a function of *ZQ*_S_. *η*_S,exp_ (red dots) and *η*_S,eq_ (blue dots) represent the efficiency calculated from the experiment and our equation, respectively. C) *ZQ*_S_ and D) the maximum generation efficiency of TE systems achieved by reducing *f*_c_ and increasing the height of TE leg (*h*), via both analyses and experiments. *η*_S,exp_ (solid dots) and *η*_S,eq_ (hollow dots) represent the efficiency calculated from the experiment and our equation, respectively.

The accuracy of Equation (4) is verified by comparing the system efficiency given by Equation (4) (*η*_S,eq_) and numerically calculated one (*η*_S,num_) by solving the governing equation (Equation S22, Supporting Information), by varying the *f*_c_ in a wide range of 0.01–10 under *q*_h_ *·h*/*F* = 100 W m^−1^ and *T*_c_ = 300 K (**Figure** **4**A). This indicates good consistency. More influence parameters over broader data ranges, including *q*_h_ *·h*/*F* = 0.1–1000 W m^−1^ and *T*_c_ = 300–450 K, can be found in Figure S4 (Supporting Information), indicating a minor error within 4% under most operating conditions.

Furthermore, we conducted experiments to investigate the effectiveness of Equation (4) using the setup shown in Figure 3E. Here, ZrO_2_ blocks of different thicknesses (3, 5, and 10 mm) were used to vary the thermal resistance on the cold side of the TEG system. The integrated average values of the material properties in the range 300–450 K were used to estimate the efficiency. The detailed parameters for each test are listed in Table S1 (Supporting Information). Figure 4B shows the linear relationship between the experimentally measured system efficiency (*η*_S,exp_) and *ZQ*_S_. For comparison, the directly predicted system efficiency given by Equation (4) is also shown. Notably, the experimental error between the *η*_S,exp_ and *η*_S,eq_ of the TE systems (Figure 4B) is inverse as compared with the *η*_D,exp_ and *η*_D,eq_ (Figure 3F). This could be attributed to the extra error introduced by the interface thermal resistance caused by ZrO_2_ blocks.

### 2.3 Optimizing Strategies for TE Systems Under Type‐II TB Condition

The linear correlation of the efficiency with the new figure of merit, *ZQ*_S_, indicates that the system efficiency can be increased by improving the *ZQ*_S_. The *ZQ*_S_ was positively correlated with the *PF*, *h*, and *q*_h_, and negatively correlated with the *κ*, *f*_c_, *F*, and *T*_c_. Among the seven parameters, *PF* and *κ* are the material properties; *h* and *F* are the structural parameters; and *q*_h_, *f*_c_, and *T*_c_ are identified as the external thermal conditions. Hence, there are three main ways to boost the system efficiency in Type‐II TB conditions according to the *ZQ*_S_. The first method is to increase the *PF* or reduce the *κ*, similar to the conclusion for the Type‐I TB condition. However, for a given Ioffe's figure of merit *Z*, a material with a lower *κ* will have a higher *ZQ*_S_, i.e., a higher efficiency, in the Type‐II TB condition. In other words, materials with intrinsically low thermal conductivities, such as SnSe and Cu_2_Se, may be better choices for Type‐II TB conditional power generation. Second, increasing *h* or reducing *F* creates a larger module thermal resistance, thereby producing a larger effective temperature difference and boosting the efficiency. The third choice could be to intensify the *q*_h_ through a thermal concentration method or reduce the *f*_c_ through elaborate heat‐exchanger designs. Figure 4A suggests that *η*_S,num_ is insensitive to *f*_c_ when *f*_c_ is smaller than a specific value. This can be easily explained by the definition of *ZQ*_S_, i.e., Equation (4d). *ZQ*_S_ approaches *ZQ*_D_ and is insensitive to *f*_c_ because the term *f*_c_ *ZQ*_D_ is significantly <*F*. In other words, it would not be economic to reduce *f*_c_ for the case *f*_c_ *ZQ*_D_ <<*F*. Figure 4C shows an increase in *ZQ*_S,_ from 0.105 to 0.108 and finally 0.135, for a Bi_2_Te_3_ device in a Type‐I condition by first reducing *f*_c_ from 0.46 to 0.19, and then enhancing *h* from 1.6 to 2.0 mm. Here, the Type‐II conditions are fixed with *q*_h_/*F* = 75 kW m^−2^ and *T*_c_ = 300 K. Finally, Type‐II conditional *η*_S,exp_ changes from 2.3% (*f*_c_ = 0.46, *h* = 1.6 mm) to 2.5% (*f*_c_ = 0.19, *h* = 1.6 mm) and finally to 3.0% (*f*_c_ = 0.19, *h* = 2.0 mm), corresponding to a 30% enhancement, as shown Figure 4D.

Referring to the earlier studies on thermoelectric generators, which were exposed to nearly constant heat flux, one or more of the *ZQ*_S_‐indicated optimization strategies were used to boost the efficiency or power density. A fill factor as low as 1/300 was used in^[^ ^29^ ^]^ under a solar intensity of 1 kW m^−2^ for a STEG with a peak efficiency of 4.6%. Meanwhile, an optical concentration ratio of 200 was adopted to intensify the solar energy up to 210 kW m^−2^ in^[^ ^10^ ^]^ to achieve a system efficiency of 7.4%. In Xu's work,^[^ ^30^ ^]^ both the module parameter optimization strategies, including increasing the leg length and reducing the fill factor, and systematic thermal design strategies, including the thermal concentration and spreading, were adopted to boost the power densities of flexible TEGs for human‐body harvesting to a record high level. Notably, the radiational heat loss is non‐negligible under relatively high hot‐side temperatures. Therefore, an optimal fill factor or heat flux generally exists in these situations, and the *ZQ*_S_‐indicated optimization strategies with respect to the module parameters and systematic thermal design should be used within reasonable ranges. In general, our study provides a simple and effective efficiency formula for thermoelectric power generation under Type‐II TB conditions. In addition, the appearance of a new figure of merit, *ZQ*_S_, provides a new guideline for the design of solar power generators, radioisotope thermoelectric generators, and wearable thermoelectric generators with Type‐II TB conditions.

## 3 Conclusion

This study deals with the thermoelectric power generation problem under the Type‐II TB condition using a bottom‐up theoretical analysis and proposes a set of explicit formulae for the maximum generation efficiency at both the device and system levels, which can evaluate the device and system performance with fairly good accuracy. Notably, two engineering figures of merit for TE devices (*ZQ*_D_) and TE systems (*ZQ*_S_) were defined as indices that directly represent the power generation performance. Both, numerical calculations and experimental data confirmed the highly linear correlations of the maximum generation efficiency with *ZQ*_D_ and *ZQ*_S_ under the Type‐II TB condition. Based on the *ZQ*_S_, optimization strategies were discussed, including the optimization of the material properties, structural parameters, and external thermal conditions. Furthermore, we experimentally verified the effectiveness of the optimization strategies based on the *ZQ*_S_, and increased the Type‐II conditional *η*_S,exp_ with 30% enhancement by reducing the *f*_c_ and increasing the *h*. The appearance of *ZQ*_D_ and *ZQ*_S_ fills the absence of figures of merit suitable for TEGs operating under the Type‐II TB, and they are expected to become one of the most commonly used indexes in the thermoelectric field, similar to the *ZT*.

## 4 Experimental Section

### Statistical Analysis

The physical properties of these materials were obtained from references. MATLAB was used to fit the cubic equation curve of the material properties related to the temperature, and to process the data to verify the accuracy of the formulae.

## Conflict of Interest

The authors declare no conflict of interest.

## Author Contributions

H.L., Y.W., and K.Z. contributed equally to this work. H.L. performed investigation, deduction, simulation, and data curation. Y.W. performed data curation and wrote the original draft. K.Z. performed data curation, reversion, and methodology. Z.H., X.W., and S.W. performed reversion and methodology. W.Z. performed supervision, editing, and validation. W.L. performed conceptualization, supervision, editing, validation, methodology, and funding acquisition.

## Supporting information

Supporting Information

## Data Availability

The data that support the findings of this study are available from the corresponding author upon reasonable request.
