# Supplementary material for: General Figures of Merit ZQ for Thermoelectric Generators Under Constant Heat‐In Flux Boundary
Source: Adv Sci (Weinh). 2023 Sep 26;10(32):2303695. doi: 10.1002/advs.202303695 (PMC10646243; doi:10.1002/advs.202303695)
Supplement: Supplementary file 1 — Supporting Information [file ADVS-10-2303695-s001.pdf]

ADVANCED SCIENCE
Open Access

Supporting Information

for *Adv. Sci.*, DOI 10.1002/advs.202303695

General Figures of Merit *ZQ* for Thermoelectric Generators Under Constant Heat-In Flux Boundary

*Huan Li, Yupeng Wang, Kang Zhu, Zhijia Han, Xinzhi Wu, Shuaihua Wang, Wenqing Zhang and Weishu Liu**

Supporting information

Note S1. Reduction method of Gao's model

Gao[1, 2] proposed a new simplified method to solve the nonlinear equation of the maximum efficiency under the type-II TB condition by using the average optimized load ratio m_{ave} between m_V at the condition of $V=V_o/2$ for and m_I at the condition of $I=I_Q/2$. m_V is derived from the below equation as follows

$$\begin{cases} \left(T_h - T_c\right)\left(1 + Z\dfrac{\left(1+2m_V\right)T_h + T_c}{2\left(1+m_V\right)^2}\right) - \Delta T_o = 0 & (a) \\ \left(T_h - T_c\right)\left(\dfrac{2m_V}{1+m_V}\right) = \Delta T_o & (b) \\ \Delta T_o = Q_h R_t & (c) \end{cases} \quad \text{(S1)}$$

Where T_h is the hot-side temperature of the TE leg, T_c is the cold-side temperature of the TE leg, ΔT_o is the temperature difference in an open circuit. m_I is derived from the below equation as follows

$$\begin{cases} \left(T_h - T_c\right)\left(1 + Z\dfrac{\left(1+2m_I\right)T_h + T_c}{2\left(1+m_I\right)^2}\right) - \Delta T_o = 0 & (a) \\ \left(T_{hs} - T_c\right)\left(1 + \dfrac{Z\left(T_{hs}+T_c\right)}{2}\right) = \Delta T_o & (b) \\ \dfrac{2\left(T_h - T_c\right)}{1+m_I} = T_{hs} - T_c & (c) \\ \Delta T_o = Q_h R_t & (d) \end{cases} \quad \text{(S2)}$$

where T_{hs} is the hot-side temperature of the TE leg when at a short circuit. Hence, m_{opt} of the maximum efficiency can be regarded as m_{ave} ,the average of m_V and m_I, and the formula can be described as follows

$$\begin{cases} \eta_{max} = \dfrac{m_{ave}}{\left(1+m_{ave}\right)^2}\dfrac{1}{\left(1+ZT_m\right)}Z\Delta T_o & (a) \\ m_{opt} = \dfrac{m_V + m_I}{2} & (b) \\ T_m = \dfrac{\left(1+2s\right)T_h + T_c}{2\left(1+s\right)^2} & (c) \\ \Delta T_o = Q_h R_t & (d) \end{cases} \quad \text{(S3)}$$

Gao provides a good idea to simplify the calculation of the maximum efficiency,

avoiding time-consuming simulation. While the application of two sets of equations (Equations S1 and S2) makes the relationship between the maximum efficiency and influence parameters (such as q_h, h, T_c, PF and κ) can't be shown directly.

Note S2. Derivation of the maximum generation efficiency formula for the TE device model under the type-II TB condition

The energy conservation equation and the heat-in flux within a single TE leg in the steady state are characterized by[3]

$$\begin{cases} \nabla \cdot \left(\kappa \nabla T \right) + \dfrac{J_\mathrm{e}^{\,2}}{\sigma} - \tau \vec{J}_\mathrm{e} \cdot \nabla T = 0 & (a) \\ \vec{q} = ST\vec{J}_\mathrm{e} - \kappa \nabla T & (b) \end{cases} \tag{S4}$$

where τ, q, J_e and are the Thomson coefficient, heat-in flux and electric current density, respectively. Assuming that the physical properties are temperature independent, the Thomson coefficient becomes zero. Thus, the governing equation and thermal boundary conditions in a one-dimensional model are given by

$$\begin{cases} \kappa \dfrac{d^2T}{dx^2} + \dfrac{I^2}{\sigma A^2} = 0 & (a) \\ Q_\mathrm{h} = ST\left(x\right)I - A\kappa \dfrac{dT}{dx}, x = 0 & (b) \\ T\left(x\right) = T_\mathrm{c}, x = h & (c) \end{cases} \tag{S5}$$

where I, A, Q_h, T_c and h are the electric current, cross-sectional area, heat input power, cold source temperature and height of the TE leg, respectively. In this section, T_c also refers to the cold-side temperature of the TE leg. The analytical solution of the governing equation and boundary conditions (Equation (S5)) leads to a temperature profile of the TE leg as

$$\begin{cases} T\left(x\right) = -\dfrac{I^2 R_\mathrm{e} R_\mathrm{t}}{2h^2} x^2 + C_1 x + C_2 \\ C_1 = \dfrac{1}{h} \dfrac{SIR_{t,I}\left(0.5I^2 R_\mathrm{e} R_\mathrm{t} + T_\mathrm{c}\right) - Q_\mathrm{h} R_\mathrm{t}}{SIR_\mathrm{t} + 1} \\ C_2 = \dfrac{0.5I^2 R_\mathrm{e} R_\mathrm{t} + T_\mathrm{c} + Q_\mathrm{h} R_\mathrm{t}}{SIR_\mathrm{t} + 1} \end{cases} \tag{S6}$$

where R_e=$h/\sigma/A$ and R_t=$h/\kappa/A$ are the electrical resistance and thermal resistance of the thermoelectric (TE) leg, respectively. κ, σ and S are the thermal conductivity,

electrical conductivity and Seebeck coefficient of TE materials, respectively. The product of S^2 and σ is called power factor (*PF*).

Then, the effective temperature difference of the TE leg is given

$$\Delta T = T_\mathrm{h} - T_\mathrm{c} = \frac{0.5 I^2 R_\mathrm{e} R_\mathrm{t} - SIR_\mathrm{t} T_\mathrm{c} + R_\mathrm{t} Q_\mathrm{h}}{SIR_\mathrm{t} + 1} \tag{S7}$$

where T_h is the heat source temperature, which also refers to the hot-side temperature of the TE leg in this section. Moreover, the electric current in the TE module is also governed by the temperature difference of the TE leg as following

$$I = \frac{S\left(T_\mathrm{h} - T_\mathrm{c}\right)}{R_\mathrm{e}\left(m+1\right)} \tag{S8}$$

where m is the ratio of the load resistance to the TE leg electrical resistance. According to Eqs. S7 and S8, the current is solved. The negative solution is unconsidered and the exact expression of the current is given as

$$I = \frac{\sqrt{\left[S^2 R_\mathrm{t} T_\mathrm{c} + (m+1) R_\mathrm{e}\right]^2 + 2(2m+1) S^2 R_\mathrm{e} R_\mathrm{t}{}^2 Q_\mathrm{h}} - S^2 R_\mathrm{t} T_\mathrm{c} - (m+1) R_\mathrm{e}}{(2m+1) S R_\mathrm{e} R_\mathrm{t}} \tag{S9}$$

Then, the output power and efficiency of the TEG can be derived as

$$P = mI^2 R_\mathrm{e} \tag{S10}$$

$$\eta = \frac{P}{Q_\mathrm{h}} = \frac{2mZR_\mathrm{t} Q_\mathrm{h}}{a_1{}^2 + a_2 + a_1\sqrt{a_1{}^2 + 2a_2}} \tag{S11}$$

where $Z=S^2R_\mathrm{t}/R_\mathrm{e}=S^2\sigma/\kappa$, $a_1=ZT_\mathrm{c}+m+1$, $a_2=(2m+1)ZR_\mathrm{t}Q_\mathrm{h}$, $R_\mathrm{t}Q_\mathrm{h}=q_\mathrm{h}\cdot h/\kappa$ and $q_\mathrm{h}=Q_\mathrm{h}/A$. To further simplify Equation (S11), we numerically compare the two parts in the denominator, $a_1{}^2+a_2$ and $a_1(a_1{}^2+2a_2)^{0.5}$ with varying $q_\mathrm{h}\cdot h$, m and T_c, as shown in Figure S5. Result suggests the ratio of the two parts is almost equal to 1, i.e., $a_1{}^2+a_2\approx a_1(a_1{}^2+2a_2)^{0.5}$. Hence Equation (S11) could be further simplified as following

$$\eta_D = \frac{mZR_\mathrm{t} Q_\mathrm{h}}{a_1{}^2 + a_2} = \frac{mZR_\mathrm{t} Q_\mathrm{h}}{\left[ZT_\mathrm{c} + (m+1)\right]^2 + (2m+1) ZR_\mathrm{t} Q_\mathrm{h}} \tag{S12}$$

The generation efficiency will achieve the maximum when $\partial\eta_\mathrm{D}/\partial m=0$. The negative solution is unconsidered and the optimal ratio of the load resistance to the TE leg

electrical resistance for the maximum generation efficiency is calculated through solve the above equation.

$$m_{\mathrm{opt,D}} = \sqrt{\left(ZT_{\mathrm{c}}+1\right)^2 + ZR_{\mathrm{t}}Q_{\mathrm{h}}} \approx ZT_{\mathrm{c}}+1 \tag{S13}$$

where ZR_tQ_h is much less than $(ZT_c+1)^2$ in most working conditions except for large $q_h \cdot h$ as shown in Figure S6. The maximum generation efficiency is obtained as follows through substitute Equation (S13) into Equation (S11).

$$\begin{cases} \eta_{\max,\mathrm{D}} = \dfrac{1}{g_{\mathrm{D}}} \dfrac{\sqrt{g_{\mathrm{D}} \cdot ZQ_{\mathrm{D}}+1}-1}{\sqrt{g_{\mathrm{D}} \cdot ZQ_{\mathrm{D}}+1}+1} & (a) \\ ZQ_{\mathrm{D}} = \left(\dfrac{ZT_{\mathrm{c}}}{ZT_{\mathrm{c}}+1}\right)\left(\dfrac{h}{\kappa}\right)\left(\dfrac{q_{\mathrm{h}}}{T_{\mathrm{c}}}\right) & (b) \\ g_{\mathrm{D}} = 1 + \dfrac{1}{2\left(ZT_{\mathrm{c}}+1\right)} & (c) \end{cases} \tag{S14}$$

g_D falls into a narrow range (about 1.2 ~ 1.5) for common TE materials[4-9] as shown in Figure S1.

In this model, the exact expression of the heat source temperature can be solved by substituting Equation (S9) into Equation (S6).

$$T_{\mathrm{h}} = \frac{(m+1)\sqrt{\left[S^2R_{\mathrm{t}}T_{\mathrm{c}} + (m+1)R_{\mathrm{e}}\right]^2 + 2(2m+1)S^2R_{\mathrm{t}}^{\,2}R_{\mathrm{e}}Q_{\mathrm{h}}} - (m+1)^2R_{\mathrm{e}} + mS^2R_{\mathrm{t}}T_{\mathrm{c}}}{(2m+1)S^2R_{\mathrm{t}}} \tag{S15}$$

Then the approximate expression of the hot-side temperature can be obtained by substituting Equation (S13) into Equation (S15).

$$T_{\mathrm{h,\,eq}} = \frac{\left(2g_{\mathrm{D}}\text{-}1\right)Q_{\mathrm{h}}R_{\mathrm{t}}}{\sqrt{g_{\mathrm{D}} \cdot ZQ_{\mathrm{D}}+1}+1} + T_{\mathrm{c}} \tag{S16}$$

The accuracy of the hot-side temperature formula was investigated by a direct comparison between the temperature given by Equation (S16) ($T_{h,eq}$) and the numerically calculated Equation (S15) (T_h) over a broad range of relevant parameters and different materials[4-9]. Figures S7A and B show surprisingly good consistency between $T_{h,eq}$ and T_h within the investigated parameters range, including $q_h \cdot h$=0.1-1000 W m^{-1} and T_c=300-450 K.

For temperature dependence materials, we proposed a mathematical iteration

method to get a more precise value of the real hot-side temperature. Detail process is shown as Figure S7C. Firstly, we assume the real hot-side temperature ($T_{h,real}$) is in the range of T_c-T_c+ΔT (T_c is a known quantity for the constant heat flux boundary). Secondly, we use the integral average properties in the range of T_c-T_c+ΔT to solve Equation (S16)to get $T_{h,eq,1}$. Thirdly, we judged whether $T_{h,eq,1}$ is in the range of T_c-T_c+ΔT. If yes, we think $T_{h,eq,1}$ can be regarded as the approximate value of $T_{h,real}$. If not, we further assume the $T_{h,real}$ is in the range of T_c-T_c+2ΔT and solve Equation (S16) to get $T_{h,eq,2}$. Then we continue to judge whether $T_{h,eq,2}$ is in the range of T_c-T_c+2ΔT, and circulate the above process until $T_{h,eq,N}$ is in the range of T_c-T_c+NΔT (Each cycle N increases by 1). In order to show the rationality of this iteration method, we compare the hot-side temperature measured by experiments (red dots) with calculation by iteration (blue dots), as shown in Figure S7D. Experimental data come from Table S1. The experimental values match well with the values obtained through iteration, proving iteration is a feasible and precise method to evaluate the hot-side temperature.

Note S3. Measuring the maximum generation efficiency of TE devices and systems under the type-II TB condition

Figure S8A illustrates the equipment designed to measure the generation efficiency of TE devices and systems under the type-II TB condition. The input power is supplied by a resistance heater. An insulation made of asbestos is used to reduce the heat loss from the upper surface of the heater. A heat flux meter made of steel is employed to measure the heat input power (Q_h) through the Fourier law. Uniform heat flux flowing into the heat flux meter is achieved by using a heat concentrator made of copper with four prisms. Each plane temperature of the heat flux meter is measured by two thermocouples (TT-T-30, Omega), and the average value is recorded by the temperature measuring terminal input module (NI-9214, National Instruments). As shown in Figure S8B, the change of heat-in flux with the external resistance during the testing process is very small, which is beneficial for accurately measuring the maximum efficiency under the type-II TB condition. ZrO_2 ceramic plates with

different thick act as the various thermal resistance between the TEG and the environments. As for the Al_2O_3 ceramic plate and copper electrode, we neglect their thermal resistance due to their much higher thermal conductivities compared to TE materials. The cold-side of the equipment is cooled by a temperature control module (a TE device controlled by the PID program) and the water-cooling system to achieve constant cold-side temperature. Thermal grease is used on the surface between each component, and external force is applied to a heat insulator to reduce contact thermal resistance. The surfaces of the heat concentrator and heat flux meter are pasted with thin aluminum foil with extremely low emissivity to reduce the heat radiation loss. The main part of the equipment is placed in a vacuum to achieve higher accuracy by reducing the influence of heat convection. Out of the vacuum chamber, a resistance box (ZX90, Shanghai Chengyang Instrument Co., Ltd) is connected in series to the circuit to adjust the load resistance. The voltage at both sides of the resistance box (V_{load}) is measured by a sourcemeter (Model 2450 System SourceMeter, Keithley Instruments). A DC regulated power supply is employed to supply current to the heater. The TE module tested is made by commercial Bi_2Te_3 (Guangdong Fuxin Technology Co., Ltd), and detailed material properties are shown in Figure S3.

Before the test, for obtaining accurate results, copper electrode resistance (R_{Co}) and wire resistance (R_{wire}) between the resistance box and TE module are measured in advance. The resistance of the wire is measured by Kelvin four-terminal sensing using the 2450 sourcemeter. The resistance of the copper electrode is calculated by Ohm law ($R_{Co}=h_{Co}/A_{Co}/\sigma_{Co}$), and the shape parameter is measured by vernier caliper and screw micrometer. Thus, the generation efficiency can be calculated as $\eta=(V_{load}/R_{load})^2\cdot(R_{load}+R_{wire}+R_{Co})$. During the test, we first adjust the resistance of the resistance box (R_{load}) to the expected value. Then, the current of the resistance heater is changed until the input power density (q_h) reaches the specified value. When the fluctuation of input power density q_h doesn't exceed 0.1 kW/m^2 during 30 minutes, we regard the TE system reaches a thermal steady state. The resistance and voltage of resistance box as well as the temperature of each measuring position are recorded. Subsequently, by changing the resistance of the resistance box and repeating the

above process, the maximum generator efficiency can be found in this way. Figure S8C and D show the change of efficiency and hot-side temperature with different external resistance.

The transport properties of commercial n-BiTe and p-BiTe used in this study have a correlation with the temperature, as shown in Figure S3. Hence, we use the integral average of the properties to estimate the efficiency, and choose an empirical temperature interval 300–450 K as the upper and lower limits of integration. We compared experimental efficiencies with prediction efficiencies calculated by properties at 300 K, the integral average properties (300-450 K) and integral average properties (300-$T_{h,real}$). Within, $T_{h,real}$ is the experimentally measured value of the hot-side temperature. The experimental data is obtained from Table S1. We found only using the constant material properties at 300 K causes a relatively higher error compared with the value calculated by integral average. And the error calculated by 300-450 K interval is similar to that of 300-$T_{h,real}$, which proves the usage of 300-450 K can also be a precise choice. Hence, we use an experiential temperature (300-450 K) as the lower and upper limit temperate of the integral average. The detailed processes of integral average are described as follows

$$\begin{cases} S_{\text{int,X}} = \dfrac{\int_{T_{\text{c,int}}}^{T_{\text{h,int}}} S_{\text{X}}(T)dT}{T_{\text{h,int}} - T_{\text{c,int}}} & (a) \\ R_{\text{e,int,X}} = \dfrac{\int_{T_{\text{c,int}}}^{T_{\text{h,int}}} R_{\text{e,X}}(T)dT}{T_{\text{h,int}} - T_{\text{c,int}}} & (b) \\ R_{\text{t,int,X}} = \dfrac{\int_{T_{\text{c,int}}}^{T_{\text{h,int}}} R_{\text{t,X}}(T)dT}{T_{\text{h,int}} - T_{\text{c,int}}} & (c) \end{cases} \tag{S17}$$

where X={n, p, $T_{h,int}$ is the upper limit temperature of integral average, $T_{c,int}$ is the lower limit temperature of the integral average, $S_{int,X}$ is the integral average of Seebeck coefficient, $R_{e,int,X}$ is the integral average of electrical resistance $R_{e,X}(T)$ and $R_{t,int,X}$ is the integral average of thermal resistance $R_{t,X}(T)$. Herein, $R_{e,X}(T)=h/A/\sigma(T)$ and $R_{t,X}=h/A/\kappa(T)$. For convenience, the n-type and p-type are regarded as a whole, and the properties of this whole can be derived as follow

$$\begin{cases} S_{\text{int}} = S_{\text{int,n}} + S_{\text{int,p}} & (a) \\ R_{\text{e,int}} = R_{\text{e,int,n}} + R_{\text{e,int,p}} & (b) \\ R_{\text{t,int}} = \dfrac{R_{\text{t,int,n}} R_{\text{t,int,p}}}{R_{\text{t,int,n}} + R_{\text{t,int,p}}} & (c) \end{cases} \tag{S18}$$

S_{int}, $R_{\text{e,int}}$, $R_{\text{t,int}}$ are used to verify the experimental result and literature result[10]. Besides, only the TE efficiency in this paper is considered, while the others, such as the optical efficiency and absorber efficiency, are not taken into account here.

Note S4. Derivation of the maximum generation efficiency formula for TE systems model under the type-II TB condition

The governing equation and boundary conditions of the TE system in the steady state are given by

$$\begin{cases} \kappa \dfrac{d^2T}{dx^2} + \dfrac{I^2}{\sigma A^2} = 0 & (a) \\ \dfrac{Q_{\text{h}}}{F} = ST(x)I - A\kappa \dfrac{dT}{dx}, x = 0 & (b) \\ ST(x)I - A\kappa \dfrac{dT}{dx} = \dfrac{T(x) - T_{\text{c}}}{f_{\text{c}} R_{\text{t}}}, x = h & (c) \end{cases} \tag{S19}$$

where F is the fill factor ($F=A_{\text{t}}/A_{\text{rec}}$), T_{c} is the cold source temperature, which does not refer to the cold-side temperature of the TE leg in this section, and f_{c} is defined as the ratio of the cold-side thermal resistance to the thermal resistance (R_{t}) of the TE module. The solving process is similar to that of the TE device model. The analytical solution of the governing equation and boundary conditions Equation (S19) leads to a temperature profile of the TE leg as

$$\begin{cases} T(x) = -\dfrac{I^2 R_{\text{e}} R_{\text{t}}}{2h^2} x^2 + C_1 x + C_2 \\ C_1 = \dfrac{1}{h} \dfrac{SIR_{\text{t}} \left[0.5 I^2 R_{\text{e}} R_{\text{t}} \left(1 - SIf_{\text{c}} R_{\text{t}} + 2 f_{\text{c}}\right) + T_{\text{c}}\right] - \dfrac{Q_{\text{h}}}{F} R_{\text{t}} \left(1 - SIf_{\text{c}} R_{\text{t}}\right)}{SIR_{\text{t}} \left(1 - SIf_{\text{c}} R_{\text{t}}\right) + 1} \\ C_2 = \dfrac{0.5 I^2 R_{\text{e}} R_{\text{t}} \left(1 - SIf_{\text{c}} R_{\text{t}} + 2 f_{\text{c}}\right) + T_{\text{c}} + \dfrac{Q_{\text{h}}}{F} R_{\text{t}} \left(1 - SIf_{\text{c}} R_{\text{t}} + f_{\text{c}}\right)}{SIR_{\text{t}} \left(1 - SIf_{\text{c}} R_{\text{t}}\right) + 1} \end{cases} \tag{S20}$$

Then, the effective temperature difference of the TE leg is given

$$\Delta T = T_{\mathrm{h}} - T_{\mathrm{c}}^{'} = \frac{0.5I^2 R_{\mathrm{e}} R_{\mathrm{t}} \left(1 - 2SIf_{\mathrm{c}} R_{\mathrm{t}}\right) - SIT_{\mathrm{c}} R_{\mathrm{t}} + \frac{Q_{\mathrm{h}}}{F} R_{\mathrm{t}} \left(1 - SIf_{\mathrm{c}} R_{\mathrm{t}}\right)}{SIR_{\mathrm{t}} \left(1 - SIf_{\mathrm{c}} R_{\mathrm{t}}\right) + 1} \tag{S21}$$

where T_{h} is the heat source temperature, which also refers to the hot-side temperature of the TE leg in this section, $T_{\mathrm{c}}^{'}$ is the cold-side temperature of the TE leg. Then, the governing model of TE systems is given

$$\begin{cases} T_{\mathrm{h}} - T_{\mathrm{c}}^{'} = \dfrac{0.5I^2 R_{\mathrm{e}} R_{\mathrm{t}} \left(1 - 2SIf_{\mathrm{c}} R_{\mathrm{t}}\right) - SIT_{\mathrm{c}} R_{\mathrm{t}} + \frac{Q_{\mathrm{h}}}{F} R_{\mathrm{t}} \left(1 - SIf_{\mathrm{c}} R_{\mathrm{t}}\right)}{SIR_{\mathrm{t}} \left(1 - SIf_{\mathrm{c}} R_{\mathrm{t}}\right) + 1} & (a) \\ I = \dfrac{S\left(T_{\mathrm{h}} - T_{\mathrm{c}}^{'}\right)}{R_{\mathrm{e}}\left(m+1\right)} & (b) \\ \eta = \dfrac{I^2 R_{\mathrm{e}} mF}{Q_{\mathrm{h}}} & (c) \end{cases} \tag{S22}$$

A cubic equation of I is generated in the numerator of Equation (S21), which makes the governing model difficult to solve analytically, so further simplification is needed to obtain the solution for the maximum generation efficiency. At first sight, the Seebeck coefficient is generally on the order of 10^{-4} V/K, thus the term $SIf_{\mathrm{c}}R_{\mathrm{t}}$ is liable to be of small values. Moreover, f_{c} is usually smaller than 1, and I always decrease with R_{t} increasing. Therefore, the terms $SIf_{\mathrm{c}}R_{\mathrm{t}}$ in the highest order of I lying the numerator and denominator are both simplified to 1 for further derivation. A similar dealing method is also shown in our group's earlier work[3]. Then, the final effective temperature difference of the TE leg is written as

$$\Delta T = T_{\mathrm{h}} - T_{\mathrm{c}}^{'} = \frac{0.5I^2 R_{\mathrm{e}} R_{\mathrm{t}} - SIT_{\mathrm{c}} R_{\mathrm{t}} + \frac{Q_{\mathrm{h}}}{F} R_{\mathrm{t}} \left(1 - SIf_{\mathrm{c}} R_{\mathrm{t}}\right)}{SIR_{\mathrm{t}} + 1} \tag{S23}$$

The current can be solved when combing the Equation (S23) and Equation (S8). The negative solution is unconsidered and the current expression is given as

$$I = \frac{\sqrt{\left[S^2 R_{\mathrm{t}}\left(T_{\mathrm{c}} + \frac{Q_{\mathrm{h}}}{F} R_{\mathrm{t}} f_{\mathrm{c}}\right) + \left(m+1\right) R_{\mathrm{e}}\right]^2 + 2\left(2m+1\right) S^2 R_{\mathrm{e}} R_{\mathrm{t}}^2 \frac{Q_{\mathrm{h}}}{F}} - S^2 R_{\mathrm{t}}\left(T_{\mathrm{c}} + \frac{Q_{h}}{F} R_{\mathrm{t}} f_{\mathrm{c}}\right) - \left(m+1\right) R_{\mathrm{e}}}{\left(2m+1\right) S R_{\mathrm{e}} R_{\mathrm{t}}} \tag{S24}$$

The generation efficiency of TE systems can also be derived as

$$\eta = \frac{P}{Q_\mathrm{h}/F} = \frac{2mZR_\mathrm{t}Q_\mathrm{h}}{a_3^2 + a_2 + a_3\sqrt{a_3^2 + 2a_2}}\frac{1}{F} \tag{S25}$$

where $a_2=(2m+1)ZR_\mathrm{t}Q_\mathrm{h}/F$ and $a_3=(ZT_\mathrm{c}+m+1)+ZR_\mathrm{t}Q_\mathrm{h}f_\mathrm{c}/F$. The form of the Equation (S25) is similar to that of Equation (S11) except for the introduction of the term $ZQ_\mathrm{h}R_\mathrm{t}f_\mathrm{c}/F$ in a_3. Thus, the maximum generation efficiency can be derived by analogy with the derivation process in the previous section. To further simplify Equation (S25), we numerically compare the two parts in the denominator, $a_3^2+a_2$ and $a_3(a_3^2+2a_2)^{0.5}$, with varying $q_\mathrm{h}\cdot h/F$, m, T_c and f_c, as shown in Figure S9. Results suggest the ratio of the two parts is almost equal to 1, i.e., $a_3^2+a_2 \approx a_3(a_3^2+2a_2)^{0.5}$. Hence Equation (S25) could be further simplified as following

$$\eta_\mathrm{S} = \frac{mZR_\mathrm{t}Q_\mathrm{h}}{a_3^2 + a_2}\frac{1}{F} = \frac{mZR_\mathrm{t}Q_\mathrm{h}}{\left[\left(ZT_\mathrm{c} + m + 1\right) + Z\frac{Q_\mathrm{h}}{F}R_\mathrm{t}f_\mathrm{c}\right]^2 + \left(2m+1\right)ZR_\mathrm{t}\frac{Q_\mathrm{h}}{F}}\frac{1}{F} \tag{S26}$$

The generation efficiency reaches the maximum value when $\partial\eta_\mathrm{S}/\partial m=0$. The negative solution is unconsidered and the optimal ratio of the load resistance to the TE module electrical resistance for the maximum generation efficiency is calculated through solving the above equation.

$$m_\mathrm{opt,S} = \sqrt{\left[\left(ZT_\mathrm{c}+1\right) + Z\frac{Q_\mathrm{h}}{F}R_\mathrm{t}f_\mathrm{c}\right]^2 + ZR_\mathrm{t}\frac{Q_\mathrm{h}}{F}} \tag{S27}$$

Noted that we have verified $ZR_\mathrm{t}Q_\mathrm{h} \ll (ZT_\mathrm{c}+1)^2$ in the previous section (Figure S6). Hecne, it is easy to prove that $ZR_\mathrm{t}Q_\mathrm{h}/F \ll [(ZT_\mathrm{c}+1)+ZQ_\mathrm{h}R_\mathrm{t}f_\mathrm{c}/F]^2$. Consequently, Equation (S27) could be simplified as following

$$m_\mathrm{opt,S} \approx \left(ZT_\mathrm{c}+1\right) + Z\frac{Q_\mathrm{h}}{F}R_\mathrm{t}f_\mathrm{c} \tag{S28}$$

The maximum generation efficiency is obtained as follows through substituting Equation (S28) into Equation (S25)

$$
\begin{cases}
\eta_{\max,S} = \dfrac{1}{g_S} \dfrac{\sqrt{g_S \cdot ZQ_S + 1} - 1}{\sqrt{g_S \cdot ZQ_S + 1} + 1} & (a) \\
ZQ_S = \dfrac{ZQ_D}{F + f_c ZQ_D} & (b) \\
g_S = 1 + \dfrac{1}{2\left[\left(ZT_c + 1\right) + Zf_c q_h h / F / \kappa\right]} & (c)
\end{cases}
\tag{S29}
$$

The g_S (Equation (S29c)) is a little lower than g_D (Equation (S14c)) due to the extra term $Zf_c q_h h/(F\kappa)$ in the denominator. Hence g_S value is also approximately a constant. Figure S10A and B show the two-dimensional plots of $\eta_{S,num}$ versus the PF (0.2-5 mW m^{-1} K^{-2}) and κ (0.5-2 W m^{-1} K^{-1}) under $q_h \cdot h/F$ =100 W m^{-1}, T_c=300K and f_c=0.1. Obviously, the contours of ZQ_S almost coincide with the contours of $\eta_{S,num}$, while the contours of g_S show poor consistency with that of $\eta_{S,num}$. g_S cannot reflect the variation of efficiency when the material properties (PF and κ) change. Thus, ZQ_S is selected as a newly defined indicator, while g_S is used as a correction coefficient.

Note that q_h, h and F always exist in the form of $q_h \cdot h/F$ in the TE system equation (Equation (S29)), while the impact of q_h/F and h is implicit in the governing equation (Equation (S22)). To prove the equivalent effect of q_h/F and h, the evolution of the efficiency is numerically calculated by the governing equation Equation (S22) ($\eta_{S,num}$) with a wide range of q_h/F (0.1-1000 kW m^{-2}) under specific sets of $q_h \cdot h/F$, including $q_h \cdot h/F$=0.1, 1, 10, 10 and 1000 W m^{-1}. As shown in Figure S11, $\eta_{S,num}$ is unchanged with q_h when $q_h \cdot h/F$ is fixed. So the influence of $q_h \cdot h/F$ is discussed as a whole.

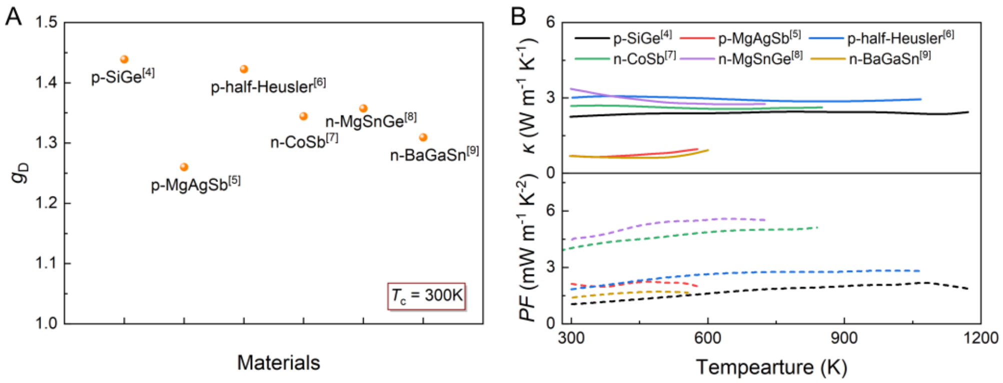

Figure S1 (A) Value of g_D=1+0.5/(ZT_c+1) for different TE materials[4-9]. It is close numerically for different TE materials. The physical properties of TE materials[4-9] at 300 K are used and the cold source temperature is 300 K. (B) Evolution of the thermal conductivity and power factor of

studied materials[4-9] with the temperature.

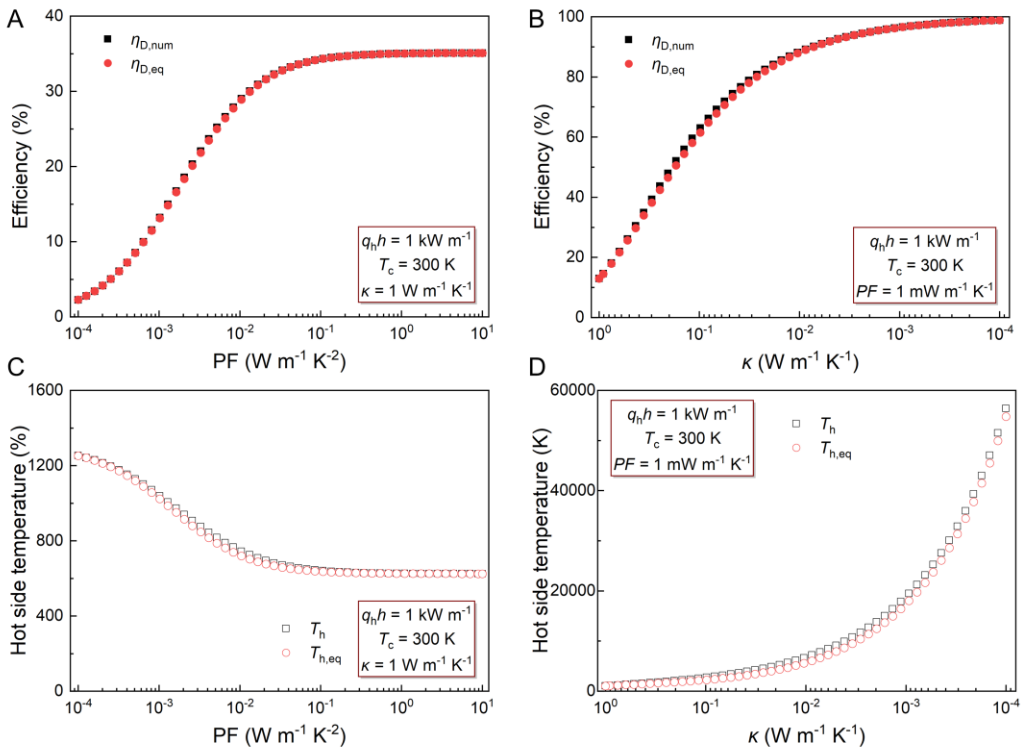

Figure S2 The maximum efficiency of the TE device under different (A) power factor (*PF*) and (B) thermal conductivity (κ). Black and red symbols represent the maximum generation efficiency numerically calculated from the governing equation ($\eta_{S,num}$) and the value calculated through our equation ($\eta_{S,eq}$), respectively. The hot-side temperature of the TE device under different (C) power factor (*PF*) and (D) thermal conductivity (κ). Black and red hollow symbols represent the hot-side temperature calculated from Equation (S15) (T_h) and the value calculated through the approximate Equation (S16) ($T_{h,eq}$), respectively.

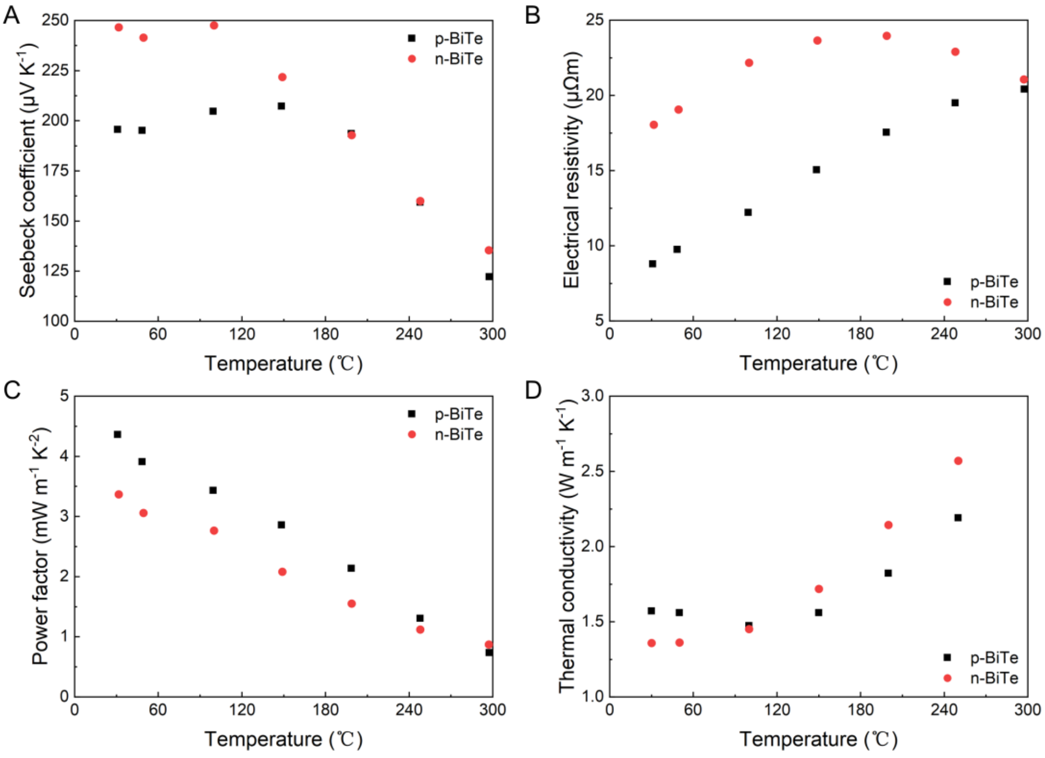

Figure S3 (A) Seebeck coefficient, (B) electrical resistivity, (C) power factor and (D) thermal conductivity of the commercial Bi_2Te_3 used in the experiments.

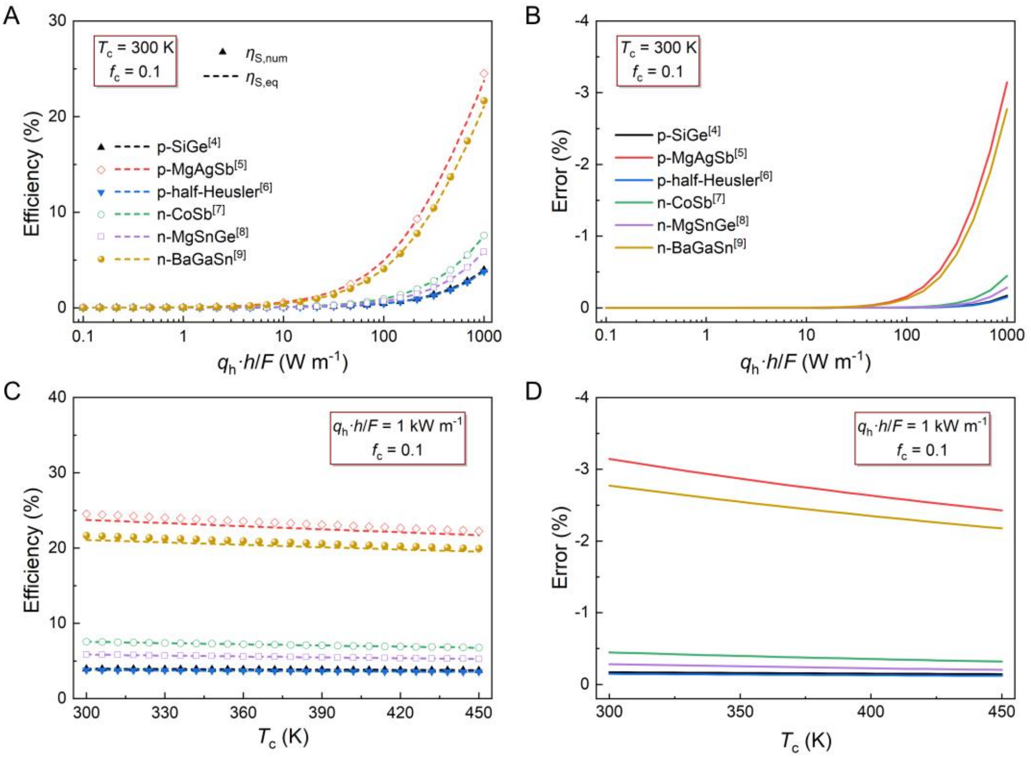

Figure S4 The maximum efficiency and its error of the TE system model under the type-II TB condition of six typical TE materials under different (A, B) heat-in flux multiplied by height of TE leg divided by fill factor ($q_h{\cdot}h/F$) and (C, D) cold source temperature (T_c). Symbols and dotted

lines represent the maximum generation efficiency numerically calculated from the governing equation ($\eta_{S,num}$) and the value calculated through our equation ($\eta_{S,eq}$), respectively. Solid lines represent the error. The physical properties of TE materials[4-9] at 300 K are used.

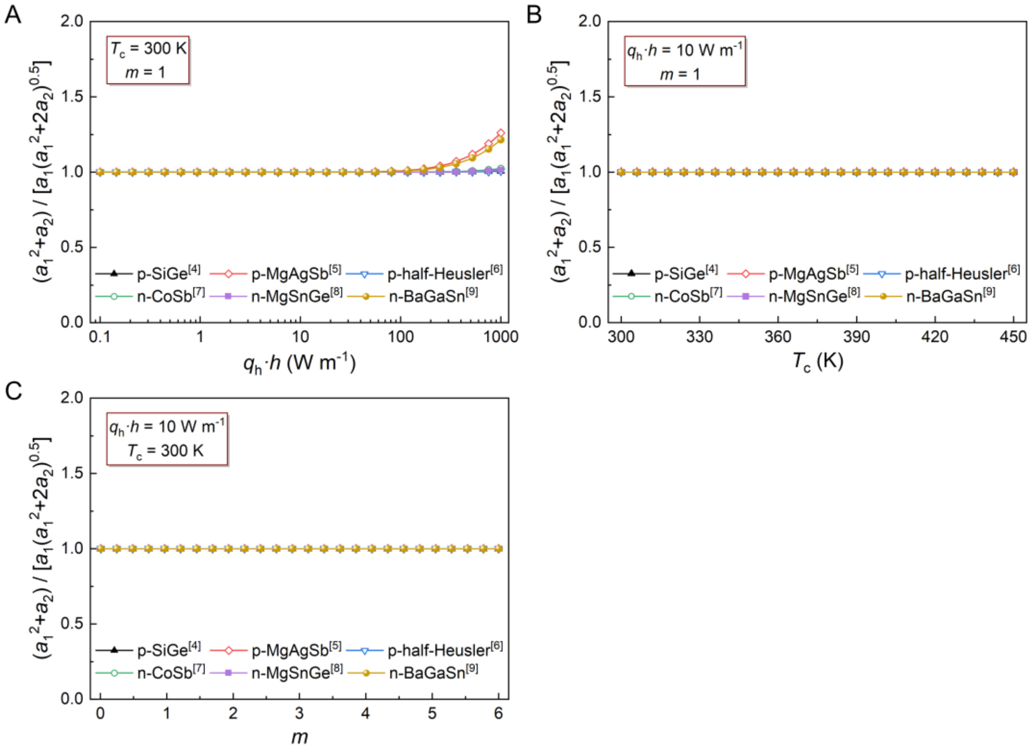

Figure S5 The ratio of $a_1^2+a_2$ to $a_1(a_1^2+2a_2)^{0.5}$ of six typical TE materials under different (A) heat-in flux multiplied by height of TE leg ($q_h \cdot h$), (B) cold source temperature (T_c) and (C) ratio of load to internal resistance (m). The value almost equals 1, so $a_1^2+a_2 \approx a_1(a_1^2+2a_2)^{0.5}$. The physical properties of TE materials[4-9] at 300 K are used.

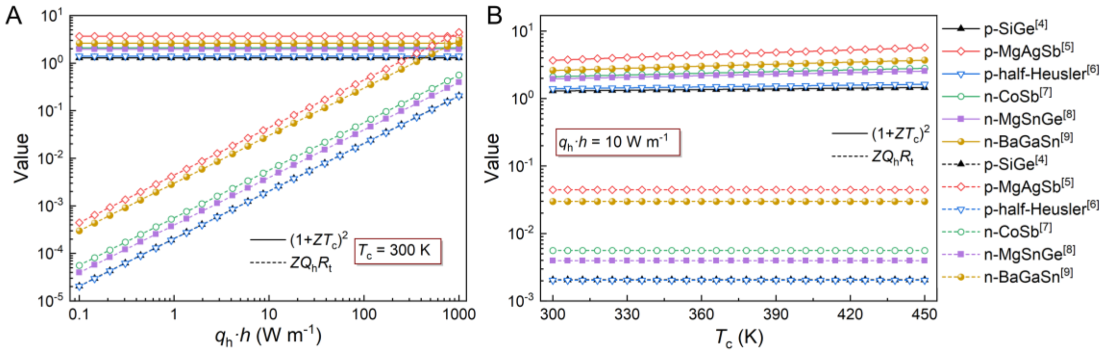

Figure S6 Value of $(1+ZT_c)^2$ and ZR_tQ_h of six typical TE materials under different (A) heat-in flux multiplied by height of TE leg ($q_h \cdot h$) and (B) cold source temperature (T_c). The physical properties of TE materials[4-9] at 300 K are used.

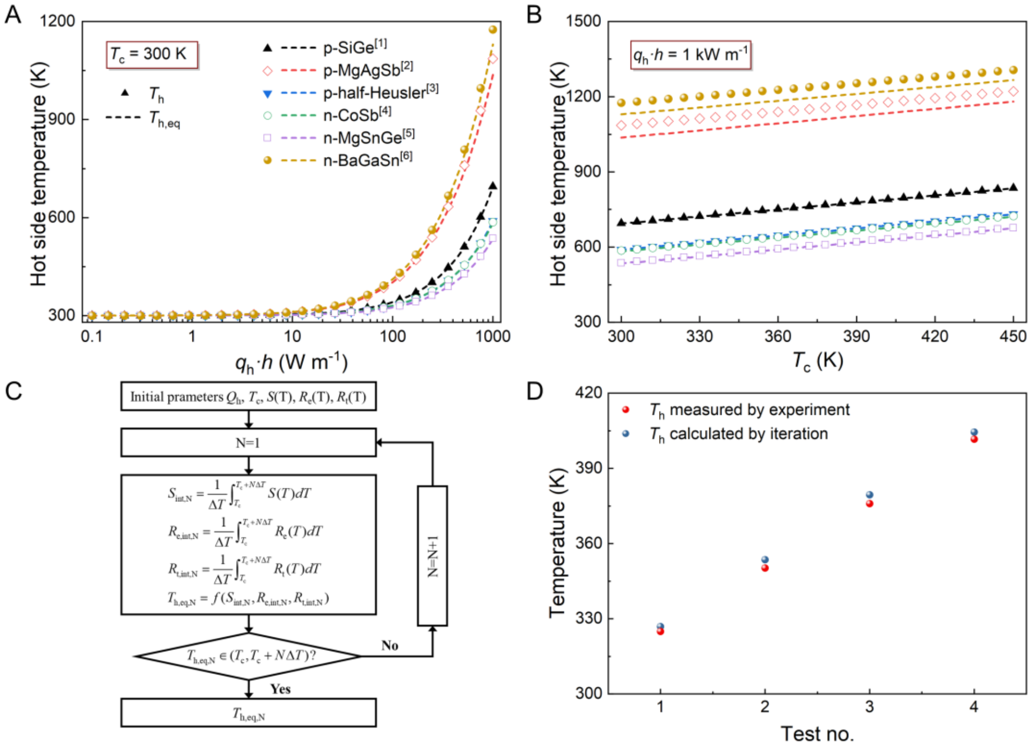

Figure S7 The hot-side temperature under the type-II TB condition of six typical TE materials under different (A) heat-in flux multiplied by height of the TE leg ($q_h \cdot h$) and (B) cold source temperature (T_c). Symbols and dotted lines represent the hot-side temperature numerically calculated from Equation (S15) (T_h) and the value calculated through our simplified Equation (S16) ($T_{h,eq}$), respectively. The physical properties of TE materials[4-9] at 300 K are used. (C) Flow chart of calculating the hot-side temperature by the iterative method. (D) Comparison of the hot-side temperature measured by experiments (red dots) with calculatyion by iteration method (blue dots).

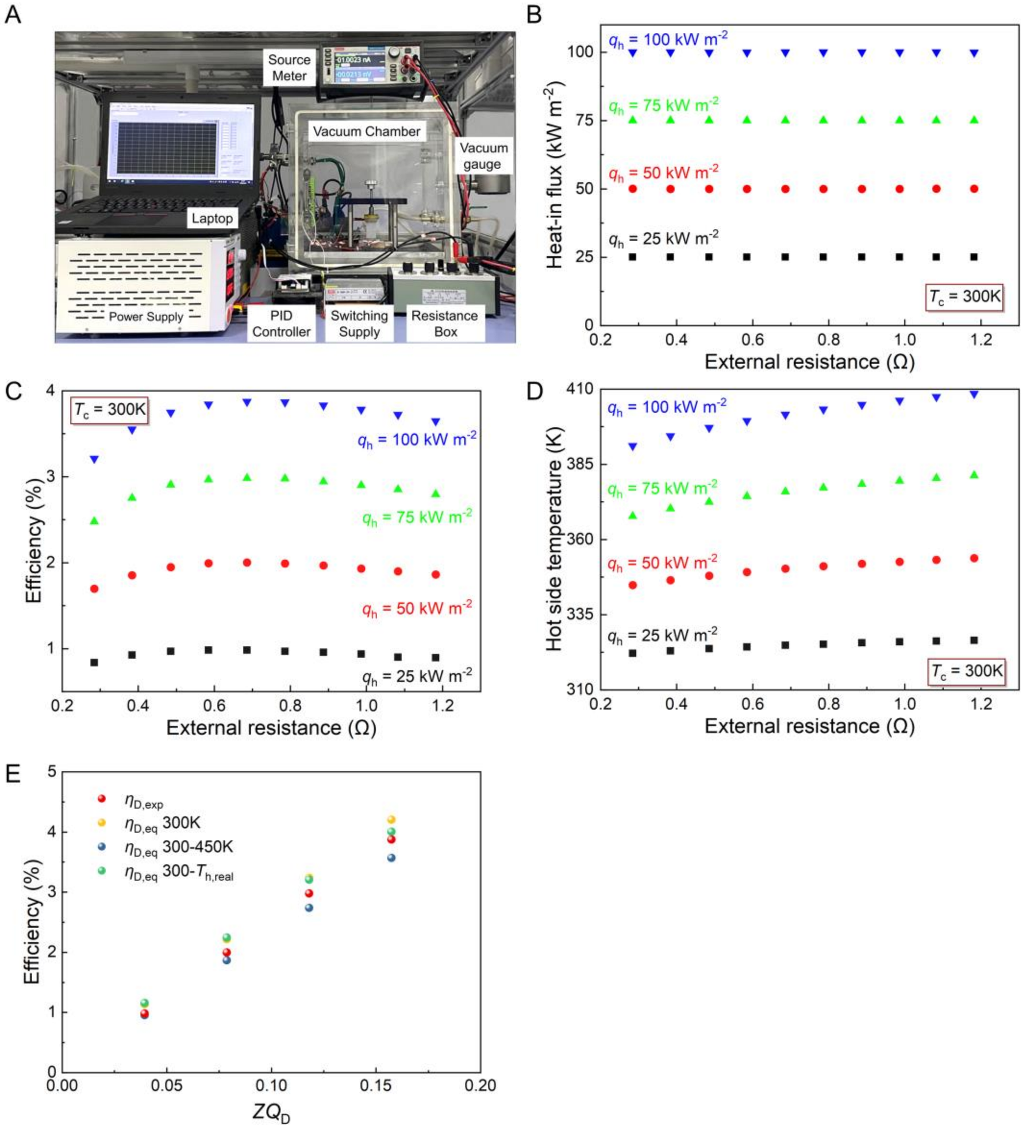

Figure S8 (A) Photo and schematic diagram for the equipment measuring the maximum generation efficiency of TE systems under the Type-II TB condition. The change of (B) heat-in flux, (C) efficiency heat-in flux and (D) hot-side temperature with external resistance during testing. (E) Comparison of experimental efficiencies (red dots) with prediction efficiencies calculated by properties at 300 K (yellow dots), the integral average properties (300-450 K) (blue dots) and integral average properties (300-$T_{h,real}$) (green dots). Within, $T_{h,real}$ is the experimentally measured value of the hot-side temperature.

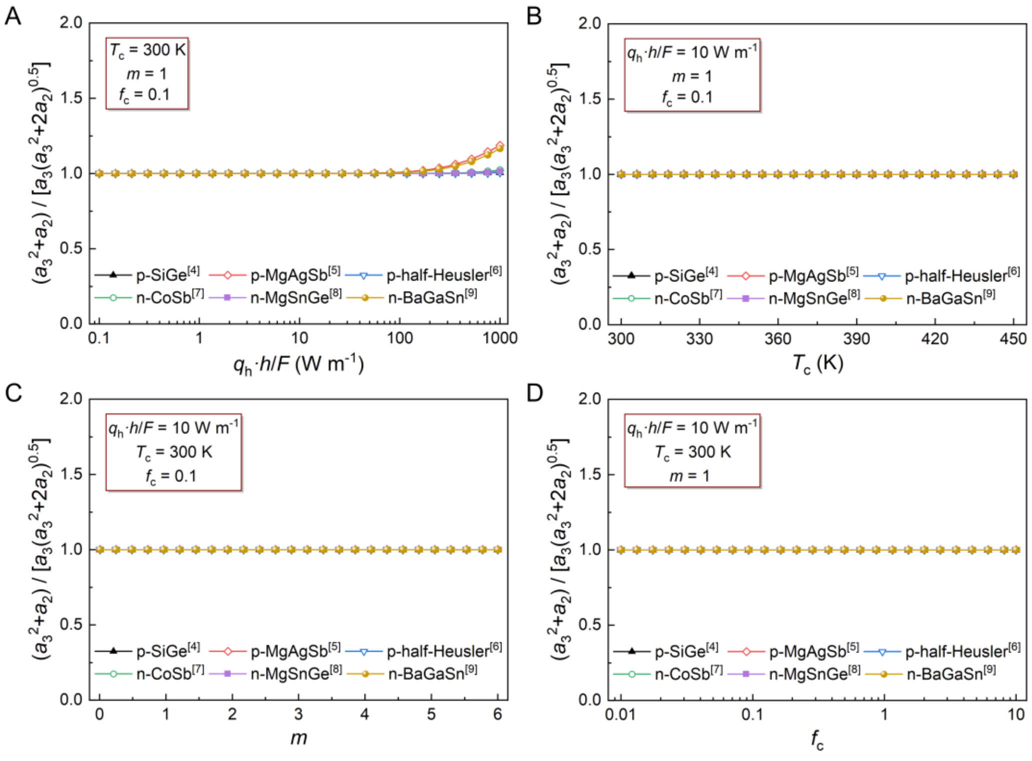

Figure S9 The ratio of $a_3^2+a_2$ to $a_3(a_3^2+2a_2)^{0.5}$ of six typical TE materials under different (A) heat-in flux multiplied by height of TE leg divided by fill factor ($q_h \cdot h/F$), (B) cold source temperature (T_c), (C) ratio of load to internal resistance (m) and (D) ratio of the cold-side thermal resistance to the thermal resistance of the TE module (f_c). The value almost equals 1, so $a_3^2+a_2 \approx a_3(a_3^2+2a_2)^{0.5}$. The physical properties of TE materials[4-9] at 300 are used.

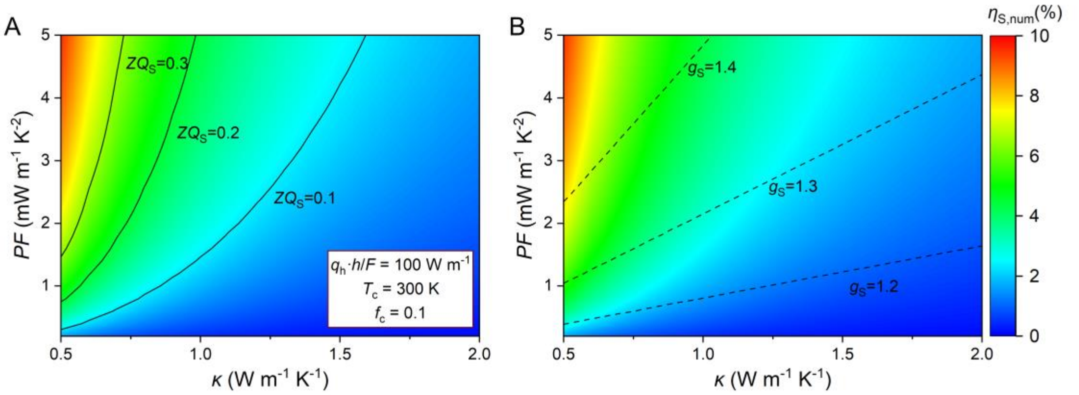

Figure S10 Two-dimensional plots of the maximum generation efficiency numerically calculated from the governing equation ($\eta_{S,num}$) versus the power factor (PF) and thermal conductivity (κ). Solid and dotted lines represent various contours of ZQ_S (A) and g_S (B) versus PF and κ, respectively.

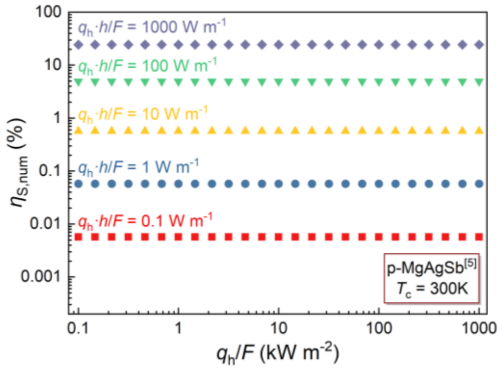

Figure S11 The maximum generation efficiency numerically calculated from governing equation ($\eta_{S,num}$) with heat-in flux divided by fill factor (q_h/F) under specific q_h/F multiplied by height of TE leg ($q_h \cdot h/F$). The physical properties of p-MgAgSb[5] are used.

Table S1. Parameters used in the TE devices during each test

Test no.	h (mm)	q_h (kW/m^2)	T_c (°C)	T_h (°C)
1	2.0	25	27	51.8
2	2.0	50	27	77.1
3	2.0	75	27	102.7
4	2.0	100	27	128.5

Table S2. Parameters used in the TE systems during each test

Test no.	f_c	h (mm)	q_h/F (kW/m^2)	T_c (°C)	T_h (°C)	T_c' (°C)
1	0.13	1.8	100	27	136.5	42.2
2	0.14	1.6	100	27	126.6	42.2
3	0.46	1.6	75	27	126.8	64.8
4	0.19	1.6	75	27	106.3	42.7
5	0.19	2.0	75	27	123.2	45.0

SI References

[1] G. Min, N. M. Yatim, *J. Phys. D*, **2008**, *41*.

[2] G. Min, *Energy Environ. Sci.*, **2022**, *15*, 356.

[3] K. Zhu, B. Deng, P. Zhang, H. S. Kim, P. Jiang, W. Liu, *Energy Environ. Sci.*, **2020**, *13*, 3514.

[4] G. Joshi, H. Lee, Y. Lan, X. Wang, G. Zhu, D. Wang, R. W. Gould, D. C. Cuff, M. Y. Tang, M. S. Dresselhaus, G. Chen, Z. Ren, *Nano Lett.*, **2008**, *8*, 4670.

[5] H. Zhao, J. Sui, Z. Tang, Y. Lan, Q. Jie, D. Kraemer, K. McEnaney, A. Guloy, G. Chen, Z. Ren, *Nano Energy*, **2014**, *7*, 97.

[6] X. Yan, W. Liu, S. Chen, H. Wang, Q. Zhang, G. Chen, Z. Ren, *Adv. Energy Mater.*, **2013**, *3*, 1195.
[7] X. Shi, J. Yang, J. R. Salvador, M. Chi, J. Y. Cho, H. Wang, S. Bai, J. Yang, W. Zhang, L. Chen, *J. Am. Chem. Soc*, **2011**, *133*, 7837.
[8] W. Liu, H. S. Kim, S. Chen, Q. Jie, B. Lv, M. Yao, Z. Ren, C. P. Opeil, S. Wilson, C. W. Chu, Z. Ren, *Proc. Natl. Acad. Sci.*, **2015**, *112*, 3269.
[9] Y. Saiga, B. Du, S. K. Deng, K. Kajisa, T. Takabatake, *J. Alloys Compd.*, **2012**, *537*, 303.
[10] D. Kraemer, B. Poudel, H. P. Feng, J. C. Caylor, B. Yu, X. Yan, Y. Ma, X. Wang, D. Wang, A. Muto, K. McEnaney, M. Chiesa, Z. Ren, G. Chen, *Nat. Mater.*, **2011**, *10*, 532.
